# Seamless site-directed mutagenesis of the *Saccharomyces cerevisiae* genome using CRISPR-Cas9

**DOI:** 10.1186/s13036-016-0028-1

**Published:** 2016-04-29

**Authors:** Damien Biot-Pelletier, Vincent J. J. Martin

**Affiliations:** Department of Biology, Concordia University, 7141 Sherbrooke West, Montréal, QC H4B 1R6 Canada; Centre for Structural and Functional Genomics, Concordia University, 7141 Sherbrooke West, Montréal, QC H4B 1R6 Canada

**Keywords:** CRISPR-Cas9, Genome editing, Site-directed mutagenesis, *Saccharomyces cerevisiae*

## Abstract

CRISPR assisted homology directed repair enables the introduction of virtually any modification to the *Saccharomyces cerevisiae* genome. Of obvious interest is the marker-free and seamless introduction of point mutations. To fulfill this promise, a strategy that effects single nucleotide changes while preventing repeated recognition and cutting by the gRNA/Cas9 complex is needed. We demonstrate a two-step method to introduce point mutations at 17 positions in the *S. cerevisiae* genome. We show the general applicability of the method, enabling the seamless introduction of single nucleotide changes at any location, including essential genes and non-coding regions. We also show a quantifiable phenotype for a point mutation introduced in gene *GSH1*. The ease and wide applicability of this general method, combined with the demonstration of its feasibility will enable genome editing at an unprecedented level of detail in yeast and other organisms.

## Letter to the editor

Following the first reported application of CRISPR-Cas9 in *Saccharomyces cerevisiae* [1], several methods exploiting the potential of this technology for yeast genome editing were published enabling gene disruption [1–4], gene deletion [5, 6], heterologous sequence integration [2, 4, 5, 7, 8], and insertion of point mutations [1, 5–7]. The genome of a strain of *Saccharomyces cerevisiae* evolved by genome shuffling for resistance to a toxic lignocellulosic hydrolysate (designated R57) was recently sequenced uncovering 17 single nucleotide variations with its parent strain [9]. We reasoned that recent developments in CRISPR-Cas9 technology should permit the seamless introduction of the point mutations discovered in R57 back into the wildtype parental background for testing phenotype to genotype associations. Similarly, we set to revert each of these point mutations to wildtype in the mutant strain R57. However, strategies reported for the introduction of a point mutation using CRISPR-Cas9 suffer several caveats that restrict the range of mutations that can be introduced at any given locus. One difficulty is that sequencing is required to detect the successful integration of a point mutation. However, the main challenge to using CRISPR-Cas9 for the introduction of point mutations is the risk of repeated cutting by Cas9 after homology directed repair (HDR) of the initial double stranded break (DSB). Indeed, point mutations may not be located within the protospacer sequence, leaving it intact after HDR. Even if the mutation is located close enough to a protospacer adjacent motif (PAM) to modify the gRNA target sequence, a single substitution is generally insufficient to prevent recognition by the gRNA/Cas9 complex [10]. Several strategies have therefore been devised to prevent Cas9 from cutting repeatedly at the site of interest. Mutation of the PAM along with the target point mutation position abolishes target recognition by the gRNA/Cas9 complex, and has allowed the successful introduction of premature stop codons [1, 6]. This strategy remains confined to cases where the PAM site mutation is either silent or deemed inconsequential. An alternative is the insertion of so-called heterology blocks in addition to the mutation of interest [7]. A heterology block consists in a number of additional silent mutations meant to abolish gRNA recognition. While heterology blocks change codon usage in an open reading frame (ORF) and may potentially affect mRNA translation, they represent a quick and convenient means of introducing point mutations. Moreover, their successful integration is easily detected by PCR. However, the concept of a silent mutation is meaningless in untranslated regions of the genome, such as non-coding RNAs and intergenic sequences.

Mans et al. [5] demonstrated successful insertion of a point mutation without altering the PAM or resorting to a heterology block. The inserted mutation eliminated a restriction site and replaced it with another, providing for easy detection of successful mutants. Sequencing revealed that several restriction positive clones displayed additional unwanted mutations, likely due to repeated cutting by Cas9. This direct strategy therefore requires the screening of several clones by sequencing – a comparatively time consuming and costly process. The authors suggest an alternative two-step strategy for the seamless site-directed mutagenesis of the yeast genome using CRISPR-Cas9, but did not demonstrate it experimentally. A similar proposition was made shortly after by Lee et al. [11]. Here, we propose three variations on this general method, and report its successful application at 17 positions across the genome of *S. cerevisiae* haploid strains CENPK113-1A, CEN.PK113-7D and the R57 mutant diploid strain [9].

**Fig. 1:**
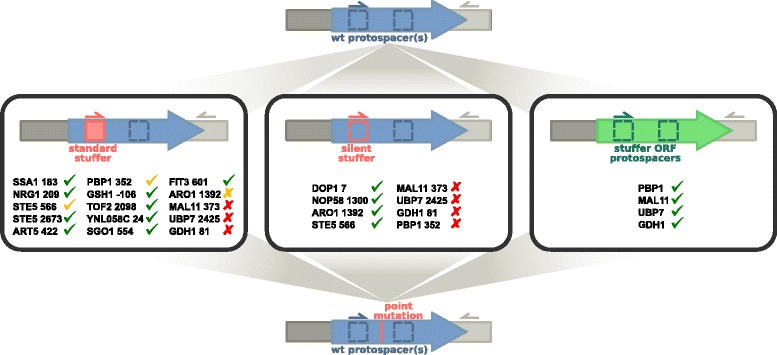
Outline of the two-step, stuffer-assisted genome site-directed mutagenesis strategy. Two variations of the strategy were applied. In the stuffer strategy a protospacer target sequence located near the site to mutagenize is replaced by a heterologous 20-nucleotide sequence (the stuffer) by CRISPR-Cas9 assisted homologous recombination, leaving the PAM site intact. The stuffer may be a standard, randomly generated sequence (the standard stuffer, *left box*) or a degenerate sequence bearing at least seven mismatches with the original protospacer (a silent stuffer, *middle box*). The second CRISPR step uses the stuffer as a protospacer, restoring the original protospacer sequence and introducing the desired mutation in a single homologous recombination event. A second variation on the strategy replaces the entire target ORF – or nearby ORF if an intergenic region is the target of mutagenesis – by a heterologous stuffer ORF (e.g. GFP), which is targeted by one or more gRNAs in the second step (*right box*). Homologous recombination restores the original ORF with mutations. Successful integration is easily assessed by PCR. The strategies were tested at the positions indicated in the boxes. Positions are identified by the coordinate of the first nucleotide of the PAM site (NGG) with respect to the nearest ORF. For each position, stuffer insertion was successful in either all strains tested (*green check*), two out of three strains (*yellow check*), in all strains but led to a slow growth phenotype (*yellow-x*), or in none of the strains (*red-x*). Once a stuffer was inserted, its removal and replacement by the point mutant sequence was successful in all cases

Using two successive CRISPR events, the method enables the introduction of point mutations without altering the PAM or inserting additional silent mutations (Fig. 1). In the first CRISPR event, the Cas9-induced DSB is repaired by a homologous repair fragment which replaces the 20 nucleotide protospacer by a heterologous sequence of the same length (termed the “stuffer”), preventing repeated cutting by Cas9 (Fig. 1). After curing of the initial guide, a second gRNA targeting the stuffer is introduced. The DSB is repaired by a DNA fragment carrying the desired point mutation, thereby removing the stuffer and abolishing recognition by the second gRNA. Stuffer insertion and removal is conveniently detected by colony PCR. This is in contrast to single-step methods that make use of sequencing to identify clones both devoid of unwanted secondary mutations, and harboring the desired point mutation, unless the point mutation coincidentally creates or removes a restriction site [5]. In the two-step method described here, the only modification introduced in the parent strain is a single point mutation (or any desired modification).

In a recent study, a similar approach was used in human induced pluripotent stem cells for the correction of heterozygous β-thalassemia mutations [12]. The *piggyBac* transposon system, carrying antibiotic resistance markers and acting as a stuffer, was inserted into the hemoglobin B gene by CRISPR assisted HDR. The transposon was then excised with the help of a specialized transposase, and the mutation corrected by homologous recombination with the non-mutant copy of the gene. Use of a two-step procedure for the seamless alteration of the yeast genome is also reminiscent of the Delitto perfetto method, whereby successive rounds of positive and negative selection are used to transiently introduce a marker cassette [13].

In the present method, a single stuffer with a random unique sequence is employed in most instances, which allows for the repeated use of the same targeting gRNA sequence and PCR primers for confirming the presence of the stuffer. For inserting or removing single point mutations, protospacer replacement was attempted for 15 out of 17 positions using the sequence 5′-agatgcgggagaggttctcg-3′ as a stuffer. Screening by PCR of three clones per position revealed that the stuffer sequence was successfully inserted in at least one of the three clones tested in all but five positions (in genes *MAL11*, *UBP7* and *GDH1* for all strains, and *STE5* 566 and *PBP1* in R57) (Fig. 1). However convenient, we suspected that the transient disruption of important genes by a standard stuffer could reduce or abolish cell viability. For example, the mutant strain R57 carries mutations in or near essential genes *DOP1* and *NOP58* that are known to be essential in *S. cerevisiae* [14, 15]. In addition, we observed that insertion of the stuffer in the *ARO1* gene of *S. cerevisiae* considerably reduced its growth rate on YPD medium (data not shown). We therefore hypothesized that failure to insert the stuffer sequence in genes *MAL11*, *UBP7*, *GDH1*, *STE5* (at position 566) and *PBP1* could be due to similar viability issues. For the two essential genes (*DOP1*, *NOP58*) and the six previously unsuccessful positions (*ARO1*, *MAL11*, *UBP7*, *GDH1*, *STE5* 566, *PBP1*), we designed custom stuffers (and stuffer targeting gRNAs) that did not disrupt the coding region using degenerate sequences. Similar to heterology blocks, our silent stuffers introduced at least seven nucleotide substitutions to protect against repeated cutting by Cas9.

We were able to insert the silent stuffers at *DOP1*, *NOP58*, *ARO1* and *STE5* (Fig. 1), and growth defects were not observed in the resulting strains. However, the silent stuffer insertion method failed for *MAL11*, *UBP7*, *GDH1* and *PBP1*. Suspecting our choice of gRNA target sequences to be the cause, we designed, for each of the four genes, two or three additional gRNAs with targets evenly spaced along the ORF to increase chances of DSBs. To avoid having to design stuffer fragments for each target, we designed donor DNAs containing the yeGFP sequence with, at their 5′ and 3′ ends, 50-bp homology to the promoter and terminator of the target genes. The expected result was the precise replacement of the native ORFs by yeGFP (Fig. 1). Not presuming of the success of any one individual guide, this strategy prevents further recognition by the gRNA/Cas9 complex anywhere in the gene by replacing the entire target ORF. The new guides were simultaneously transformed into yeast with the yeGFP stuffer. Integrants were identified in all four loci (Fig. 1), suggesting at least one guide per locus was functional. We suggest that stuffer ORFs can prove useful when the selection of a functional gRNA target is problematic. However, we note that it is not suitable in genes that are essential or strongly affect viability when deleted, in both cases preventing downstream transformation and CRISPR events.

**Fig. 2:**
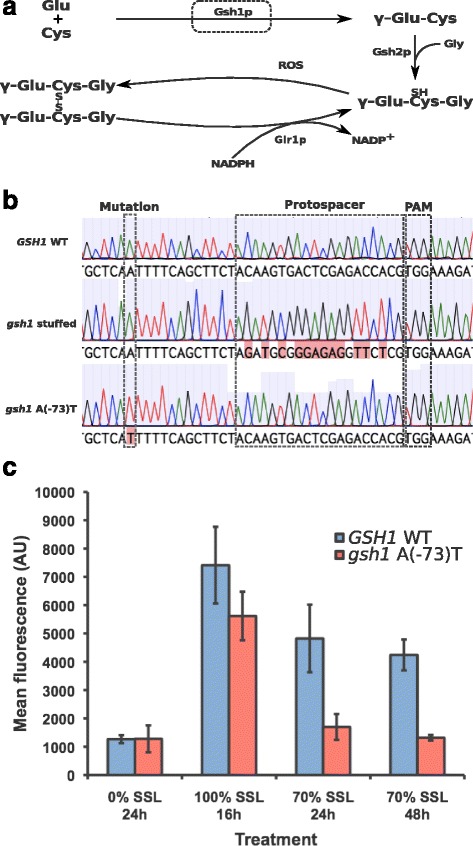
Point mutations introduced with the stuffer-assisted genome site-directed mutagenesis method lead to detectable phenotypic changes. **a** Simplified representation of glutathione synthesis and recycling. Condensation of glutamate and cysteine by Gsh1p is followed by the addition of a glycine by Gsh2p, yielding reduced glutathione. Glutathione oxidized by reactive oxygen species (ROS) is recycled to its reduced form by the NADPH-dependent Glr1p enzyme. **b** Sequencing shows successful insertion of the stuffer and subsequent introduction of a point mutation in the *GSH1* promoter sequence **c** ROS accumulation induced by exposure to SSL was compared between a *gsh1*(A(−73)T) point mutant generated with the method, and in its parent wildtype strain (WT). ROS accumulation was assessed using flow cytometry, measuring the mean fluorescence of cells treated with CellROX Deep Red reagent. ROS were measured 16 h after inoculation in minimal medium (Mid-log), after overnight incubation in undiluted SSL (acute stress), or after 24 and 48 h in minimal medium containing 70 % SSL

In strains containing the short stuffers, the second CRISPR event used DNA fragments averaging 500 bp for DSB repair and introduction of point mutations. Longer fragments spanning the promoter, ORF and terminator were required at loci stuffed with yeGFP. For all stuffer-containing strains, replacement of the stuffed sequence by the point mutant sequence was successful (Fig. 1). Introduction of points mutations was confirmed by Sanger sequencing revealing no additional unwanted mutations in the targeted loci (see Fig. 2b for an example). While the efficiency of stuffer insertion was highly variable and rarely at 100 %, we observe that for most positions considered, all clones screened for stuffer removal and point mutation insertion were positive. CRISPR efficiency was high at positions bearing the standard short and yeGFP stuffers, but lower on average for positions carrying the custom silent stuffers (data not shown). These observations suggest that a standard stuffer is useful in reducing the variability of recognition and cutting by the gRNA/Cas9 complex during the second CRISPR event. Whenever feasible, we propose that it should be the preferred method for CRISPR assisted genomic insertion of point mutations.

To demonstrate the usefulness of our seamless stuffer-assisted site-directed mutagenesis method, we chose to investigate the effect of a point mutation localized in the promoter region of gene *GSH1*, which is responsible for the committed step in glutathione biosynthesis (Fig. 2a and [16, 17]). Because the synthesis of reduced glutathione and the recycling of its oxidized form play a major role in resistance to oxidative stress (Fig. 2a), we hypothesized that a mutation of the *GSH1* promoter would modulate glutathione synthesis in the cell and thereby affect levels of reactive oxygen species (ROS). Since the mutant strain R57 was selected for its resistance to lignocellulosic hydrolysate spent sulfite liquor (SSL), we used the fluorescent CellROX Deep Red Reagent (Life Technologies) and flow cytometry to assess cytosolic ROS accumulation in wildtype and *gsh1 A(−73)T* cells upon exposure to SSL. When grown in non-toxic medium (YNB 1 % glucose), the wildtype and mutant stains accumulate comparably low levels of ROS (Fig. 2c). Subsequent exposure to undiluted SSL similarly increases ROS levels in both strains. However, following acute stress induction in 100 % SSL and transfer to YNB 1 % glucose supplemented with 70 % SSL, the mutant accumulates markedly lower amounts of ROS after 24 and 48 h incubation, suggesting the *gsh1 A(−73)T* mutation affects cell response to oxidative stress.

In the current study, we report on a strategy to introduce precise changes at the single nucleotide level in the genome of *S. cerevisiae* and demonstrate the value of the method by introducing a point mutation in the promoter region of GSH1, which leads to a measurable phenotypic effect. We believe that this two-step procedure can be applied to any organism with suitable HDR machinery at virtually any genomic coordinates to modify coding and non-coding sequences, in essential and non-essential genes. Furthermore, it is not constrained by the precise location and sequence of the PAM and protospacer. The method is less disruptive than similar two-step methods reported previously [11, 13], because it does not require the introduction of large transposons or selection cassettes. Rather, it transiently introduces few potentially silent mutations. However, the implementation of the method we have presented requires the generation of an intermediate stuffed mutant, submitted to a second cycle of transformation, PCR verification, sequencing and gRNA curing. Welcome improvements would allow stuffer integration and removal from a single transformation using for example transient or inducible gRNAs.

Because of its wide applicability, we believe this seamless, genome-level site-directed mutagenesis procedure will prove useful to a wide range of researchers interested in the precise genome editing of *S. cerevisiae* and other organisms.

## Abbreviations

CRISPR: clustered regularly interspaced short palindromic repeats
HDR: homology directed repair
PAM: protospacer adjacent motif
ORF: open reading frame
gRNA: guide RNA
DSB: double-stranded break
ROS: reactive oxygen species
SSL: spent sulfite liquor
YNB: yeast nitrogen base

## References

[1] DiCarlo JE, Norville JE, Mali P, Rios X, Aach J, Church GM (2013). Genome engineering in *Saccharomyces cerevisiae* using CRISPR-Cas systems. Nucleic Acids Res.

[2] Ryan OW, Skerker JM, Maurer MJ, Li X, Tsai JC, Poddar S, Lee ME, DeLoache W, Dueber JE, Arkin AP, Cate JHD: Selection of chromosomal DNA libraries using a multiplex CRISPR system. Elife. 2014; doi: 10.7554/eLife.03703 10.7554/eLife.03703 PMC4161972 25139909

[3] Bao Z, Xiao H, Liang J, Zhang L, Xiong X, Sun N, Si T, Zhao H (2014). Homology-integrated CRISPR-Cas (HI-CRISPR) system for one-step multigene disruption in *Saccharomyces cerevisiae*. ACS Synth Biol.

[4] Stovicek V, Borodina I, Forster J (2015). CRISPR–Cas system enables fast and simple genome editing of industrial *Saccharomyces cerevisiae* strains. Metab Eng Commun.

[5] Mans R, van Rossum HM, Wijsman M, Backx A, Kuijpers NGA, van den Broek M, Daran-Lapujade P, Pronk JT, van Maris AJA, Daran J-MG: CRISPR/Cas9: a molecular Swiss army knife for simultaneous introduction of multiple genetic modifications in *Saccharomyces cerevisiae*. FEMS Yeast Res. 2015; doi: 10.1093/femsyr/fov004 10.1093/femsyr/fov004 PMC4399441 25743786

[6] Jakočiūnas T, Bonde I, Herrgård M, Harrison SJ, Kristensen M, Pedersen LE, Jensen MK, Keasling JD (2015). Multiplex metabolic pathway engineering using CRISPR/Cas9 in *Saccharomyces cerevisiae*. Metab Eng.

[7] Horwitz AA, Walter JM, Schubert MG, Kung SH, Hawkins K, Platt DM, Hernday AD, Mahatdejkul-Meadows T, Szeto W, Chandran SS, Newman JD (2015). Efficient multiplexed integration of synergistic alleles and metabolic pathways in yeasts via CRISPR-Cas. Cell Syst.

[8] Ronda C, Maury J, Jakočiu̅nas T, Baallal Jacobsen SA, Germann SM, Harrison SJ, Borodina I, Keasling JD, Jensen MK, Nielsen AT (2015). CrEdit: CRISPR mediated multi-loci gene integration in *Saccharomyces cerevisiae*. Microb Cell Fact.

[9] Pinel D, Colatriano D, Jiang H, Lee H, Martin VJ (2015). Deconstructing the genetic basis of spent sulphite liquor tolerance using deep sequencing of genome-shuffled yeast. Biotechnol Biofuels.

[10] Fu Y, Foden J, Khayter C, Maeder ML, Reyon D, Joung JK, Sander JD (2013). High-frequency off-target mutagenesis induced by CRISPR-Cas nucleases in human cells. Nat Biotechnol.

[11] Lee ME, DeLoache WC, Cervantes B, Dueber JE (2015). A Highly Characterized Yeast Toolkit for Modular, Multipart Assembly. ACS Synth Biol.

[12] Xie F, Ye L, Chang JC, Beyer a. I, Wang J, Muench MO, Kan YW: Seamless gene correction of β-thalassemia mutations in patient-specific iPSCs using CRISPR/Cas9 and piggyBac. Genome Res*.* 2014; doi: 10.1101/gr.173427.114 10.1101/gr.173427.114 PMC4158758 25096406

[13] Storici F, Lewis LK, Resnick MA (2001). In vivo site-directed mutagenesis using oligonucleotides. Nat Biotechnol.

[14] Pascon RC, Miller BL (2000). Morphogenesis in *Aspergillus nidulans* requires Dopey (DopA), a member of a novel family of leucine zipper-like proteins conserved from yeast to humans. Mol Microbiol.

[15] Gautier T, Bergès T, Tollervey D, Hurt E (1997). Nucleolar KKE/D repeat proteins Nop56p and Nop58p interact with Nop1p and are required for ribosome biogenesis. Mol Cell Biol.

[16] Kistler M, Maier K, Eckardt-Schupp F (1990). Genetic and biochemical analysis of glutathione-deficient mutants of *Saccharomyces cerevisiae*. Mutagenesis.

[17] Ohtake Y, Yabuuchi S (1991). Molecular cloning of the gamma-glutamylcysteine synthetase gene of *Saccharomyces cerevisiae*. Yeast.

